# Risks and Protective Factors of the Prodromal Stage of Psychosis: A Literature Review

**DOI:** 10.7759/cureus.8639

**Published:** 2020-06-15

**Authors:** Aldanah Althwanay, Nada A AlZamil, Omar Y Almukhadhib, Shahd Alkhunaizi, Reem Althwanay

**Affiliations:** 1 Internal Medicine, Imam Abdulrahman Bin Faisal University, Khobar, SAU; 2 Psychiatry, Imam Abdulrahman Bin Faisal University, Dammam, SAU; 3 Medicine, Imam Abdulrahman Bin Faisal University, Dammam, SAU; 4 Emergency Medicine, King Fahd Hospital of the University, Dammam, SAU; 5 Internal Medicine, Imam Abdulrahman Bin Faisal University, Dammam, SAU

**Keywords:** psychosis, prodromal psychosis, psychotic disorders

## Abstract

Psychosis is a syndrome characterized by features of reality distortion such as delusions and hallucinations. It may occur as a primary mental disorder or secondary to a medical or neurological illness or substance abuse. Several genetic, environmental, and protective risk factors have been identified and require further study. Neurobiological damage at the onset of schizophrenia is the most active and destructive. Therefore, it is important to detect the prodromal phase of psychosis so that interventions can be started early and the onset of psychosis delayed. Herein, we review the relevant epidemiological data on psychosis, particularly in Saudi Arabia. In addition, the risk and protective factors of psychosis will be discussed.

Recent findings have shown that psychosis development is affected by genetic and environmental factors. Psychotic disorders are considered a cause of disability and are, therefore, a substantial economic burden. Consequently, it is important to try and detect the psychosis in its prodromal stage, where intervention may slow its progression and improve general wellbeing. Several tools have been identified to screen for the prodrome of psychosis, one of which is the prodromal questionnaire-brief version. This has been shown to be a promising tool that can be self-administered by the patient in contrast to long interview-based tools, which are time-consuming and require a physician to perform. Despite the limited evidence in the literature, there have been significant improvements in the outcomes of patients with psychosis when treated in the prodromal period.

In summary, this article provides psychiatrists and researchers with an overview of psychosis, its risk factors, the prodromal stage of psychosis, tools to detect the prodromal phase, and potential treatments during this phase.

## Introduction and background

Psychotic disorders are characterized by a disconnection from reality. Psychosis by itself is a syndrome rather than an illness, and it is defined as "the experience of loss of contact with reality that is not part of the person's cultural or religious beliefs." Psychosis is a sign of mental illnesses that is a diagnosis of exclusion after ruling out secondary causes, such as substance abuse (marijuana) and other illnesses, which might lead to psychosis such as stroke, some types of epilepsy, and brain tumors. Schizophrenia is the most common diagnosis when psychosis has occurred; it has three categories of symptoms: positive, which encompasses psychosis as the main feature, negative, and cognitive [1-3].

Psychotic disorders are associated with significant morbidity and disability, and they are perceived as an evolving brain pathology. Due to brain functional plasticity and adaptation, it is hoped that the early detection of psychosis may prevent disease progression [4]. Thereby, several tools were created to detect the prodromal stage of psychosis, which is defined as preliminary signs and symptoms that do not fulfill the characteristic criteria of the disease [5].

As a complex multifactorial disease, the risk factors can be partitioned into several domains, such as familial, environmental, and psychological [6]. This complex interplay provides an insight into the role of a positive environment and support system in protecting genetically susceptible individuals [7].

Herein, we review the updates of the relevant topic epidemiological data on psychosis, particularly in Saudi Arabia, and discuss the risks and protective factors of psychosis. Although this topic has been discussed extensively in several separate books and articles, each had discussed it by focusing on limited aspects and areas. Hence, we intended to review the available resources, collect all the ideas and theories, and summarize them in our review, providing wider coverage of the topic. It is hoped that this will help in the early detection and treatment of psychosis during the prodromal stage.

## Review

Definition of the prodromal stage of psychosis

The prodromal stage of schizophrenia was first conceptualized in 1911 by Bleuler and was defined as the preliminary signs and symptoms of an illness that does not fulfill the characteristic criteria of the disease [5]. It was defined by Loebel et al. as the time interval from the onset of unusual behavioral symptoms to the onset of psychotic symptoms [8].

The prodromal stage of psychosis can be detected at an early stage through the regular follow-up of high-risk patients to prevent or delay the onset of psychotic disorders. Early management of psychosis leads to better function preservation and, therefore, better long-term outcomes [8-9].

There are three subtypes of prodromal symptoms: brief intermittent psychotic syndrome (BIPS), attenuated positive symptom syndrome (APSS), and genetic risk and deterioration syndrome (GRDS) [9].

Among the general population, psychotic-like symptoms may be experienced by 20% of adults who do not fulfill the criteria for psychotic disorders [10]. Furthermore, according to Cannon et al., 16%-35% of individuals who were diagnosed with ultra-high-risk (UHR) syndrome had developed a psychotic disorder, on average, two to two and half years after recognition [11].

Tools for the detection of prodromal psychosis

Many studies have attempted to identify the early signs of psychotic disorders and mood disorders, as early detection and management may improve symptoms, decrease the rate of deterioration and relapses, and improve quality of life overall [1].

Several tools quantitatively assess and rate the severity of prodromal symptoms of psychosis, including Bonn scale for the assessment of basic symptoms (BSABS; Gross et al., 1987), the structured interview for prodromal symptoms (SIPS), the scale of prodromal symptoms (SOPS; McGlashan, 1996), the multidimensional assessment of psychotic prodrome (Yung and McGory, 1996), and the comprehensive assessment of ARMS (CAARMS; Yung et al., 2005) [12-16].

In 2011, Lowey et al. created the prodromal questionnaire - brief version" (PQ-B), which consists of 21 questions to quantitatively assess the level of prodromal psychosis in an ultra-high risk group (UHR). This was accompanied by a structured interview for prodromal syndromes (SIPS). ThePQ-B was found to have a statically significant correlation with SIPS (P<.0001). Furthermore, the difference in the total scores between the case and control groups was statistically significant [14]. This is a new promising tool in the field that relies on self-reported symptoms instead of individualized interviews, as in the previously mentioned tools. However, only a few studies have used it as their primary tool to assess prodromal psychosis, none of which were conducted in Saudi Arabia.

Xu et al. performed a pilot study among Chinese help-seeking individuals, assessing the psychometric properties of the PQ-B. In their study, the total PQ-B scores were significantly lower in the non-psychotic group than in the prodromal and psychotic groups, with p-values of <0.001 and <0.001, respectively. After a two-year follow-up, 23.9% of the prodromal group had psychosis [9].

Effects of early treatment during the prodromal stage of psychosis

The concept of early intervention in patients with a prodromal risk for psychosis was not introduced until the 1990s. Before that, no one had considered using antipsychotics for prodromal psychosis for several reasons. First, antipsychotics were known only to treat positive symptoms of schizophrenia, and it was not thought that their early use would delay or reduce relapses of the disease. Second, antipsychotics have many undesired side effects. Third, the prodromal symptoms of psychosis were not well-identified to a level that could be used for deciding when to start treatment. Fourth, the diagnosis of psychotic disorders is by exclusion. As such, its diagnosis is delayed, as other causes need to be ruled out first, and this needs to be carefully done to avoid any unnecessary stigma of such diseases [17].

There are a variety of non-specific signs and symptoms that may precede a first psychotic episode and have no specific biological markers. This means that starting treatment, even with insufficient evidence, may be an option [18].

Two clinical trials have provided evidence for early intervention in prodromal psychosis. The first was by Fallon (in 1992), who used home-based stress management techniques to identify high-risk individuals for psychosis by their primary health care physician. The therapy prevented psychosis and caused a reduction in the incidence of schizophrenia among the sample. This study had limited significance due to the small sample size [19]. The second trial was by McGorry et al. in 2002, which compared two treatments approaches among two groups who they have identified as UHR individuals: (1) the on need-based intervention (NBI), consisting of supportive psychotherapy and the possible use of antidepressants if indicated, and (2) the specific therapy intervention (SPI), consisting of low dose risperidone and cognitive behavioral therapy (CBT). The initial follow-up results after six months showed a significant difference between the two treatment groups favoring the SPI in terms of progression to psychosis. After the first six months, both groups were following NBI only; hence, the second follow-up in 12 months did not show any significant differences between both groups in terms of progression to psychosis. However, when the survival analysis is extended, taking into account levels of antipsychotic drug adherence, a significant difference is maintained between the fully adherent SPI group and the NBI group throughout follow-up. The number needed to treat for the end of treatment was 4 at the 95% confidence interval. This means that four patients would need to be treated to prevent one from progressing to psychosis over a six-month period, indicating that the intervention is relatively potent [20]. However, a most recently published article disagrees, as the administration of antipsychotics to UHR patients is potentially harmful, with no preventive benefits [21]. 

Nevertheless, Addington et al. suggested that CBT is the promising therapy for the prodromal stage of psychosis where the symptoms are minimal. On the other hand, antipsychotics seem to be beneficial in the later stages of the prodrome, where patients are on the edge to fully convert to psychosis [22].

These controversial results on whether to use antipsychotics in the prodromal stage or not mandate further trials to reach a fair conclusion. 

Epidemiology and demographics of mental health and psychotic disorders globally and in Saudi Arabia 

The World Health Organization (WHO) estimated that one in four people worldwide has a mental illness. Mental illnesses account for 25% of all disabilities in more developed countries. Moreover, the WHO considers mental illnesses as the leading cause of disability, and they are, therefore, a considerable economic burden [23]. It has also been found that, generally, mental health disorders do not occur on their own and are associated with other psychiatric disorders [24].

The incidence of first-episode psychosis varies widely between different locations but was found to be 42.6/100,000 person-years and 34/100,000 person-years globally. The risk decreases with age, and incidence rates were highest for males and females below 20 years old. There was increased risk among males and certain ethnic groups. Black and mixed ethnicities had an elevated risk and Asians a decreased risk when compared to a white British group. The risk was also increased for people with low socioeconomic status and in more urban and deprived neighborhoods [25-26].

According to the WHO, schizophrenia affects more than 23-million people worldwide, with a prevalence of 1% worldwide [27-28]. Despite the few publications about the prevalence of mental health issues in Saudi Arabia, in 2008, the Saudi Arabian Ministry of Health reported that 22.4% of mental health outpatients were diagnosed with schizophrenia or schizotypal personality and delusional disorders [29].

The median duration of untreated psychosis (DUP) in Saudi Arabia is 1.41 years. It usually takes longer for patients to seek help from psychiatric services in Saudi Arabia than it does in Western countries. This suggests that the duration of untreated psychosis is influenced by both demographic factors and pathways to care [30].

Risk factors for psychosis

According to the WHO, a risk factor is any attribute, characteristic, or exposure that increases the likelihood of developing a disease or injury by an individual. Therefore, a risk factor for psychosis should increase the likelihood of developing psychosis [31]. The risk factors of psychosis can be partitioned into several domains such as familial, environmental, and psychological [6]. For complex multifactorial disease, it seems likely that the co-exposure of two risk factors will have a greater than an additive relationship to disease risk [32].

There is a strong genetic association with psychosis. Genome-wide association studies (GWAS) revealed that several single nucleotide polymorphisms (SNPs) within the ZNF804A gene were strongly associated with schizophrenia. A relatively specific association between a common variation in GABA receptor genes and the occurrence of rare copy number variants (CNVs) has been shown to be increased in schizophrenic patients as compared with controls [33]. Establishing such genetic associations with psychosis is vital as those high-risk SNPs may be used as biomarkers for screening UHR individuals and identifying them earlier.

Studies have shown that the offsprings of a parent with psychosis are likely to be exposed to elements of the family environment that have been implicated in the development of psychotic disorders, e.g., maladaptive parental communication and practices in early childhood. As such, heritability likely influences the development of psychosis by way of gene-environment interactions, rather than through genetics alone. There is evidence that disrupted parenting through separation or death increases the risk of psychosis independently of other risk factors. A meta-analysis by Bailey et al. suggested that different aspects of childhood traumas such as sexual abuse, physical abuse, emotional abuse, and neglect have significantly impacted the development of positive psychotic symptoms such as hallucinations and delusions as well as negative symptoms [6,34].

Unfortunately, there is limited early developmental data on factors such as obesity, diet, and physical inactivity in individuals who later develop psychosis. Some research has suggested that patients with psychosis, on average, have a history of delayed motor development in childhood and adolescence as well as delayed neurological and cognitive development. Physical activity levels were found to be lower among children and adolescents who later developed non-affective psychosis [35]. Both childhood trauma and cannabis use were significantly associated with an increased risk of functional psychosis. Neurotic personality also contributed independently to this risk. These findings might help to improve the prevention of psychosis and aid in the development of specific treatment strategies [36].

Despite the strong genetic association with psychosis, recent studies, including monozygotic twin concordance studies, have found that psychosis is influenced even more by the environment, as the nature of the environment and a person's lifestyle do affect and interact with gene expression. Environmental risk factors can be divided into the following stages: (1) early life risk factors, which include prenatal and perinatal complications; (2) childhood risk factors, which include head injuries and child abuse; and (3) later-life risk factors, which include drug abuse and negative life events associated with trauma [37].

The risk factors of psychosis were divided based on their onset of assessment into (1) baseline risk factors, which could be assessed throughout the life of the individual and include (a) genetic profile, as a person who has a first-degree relative with psychosis has an increased risk for psychosis and psychotic disorders, and (b) the sex of an individual, as males have an increased risk; (2) distal risk factors that are assessed distally to the onset of illness such as (a) abnormal fetal environment and (b) abnormal cognitive development; proximal risk factors that are assessed proximally to the onset of illness and include: (a) prodromal psychotic symptoms, (b) biomarkers such as elevated dopamine levels in the brain and decreased cortical size, which can be assessed using modern neuroimaging techniques [38].

Impairment of social function was significantly associated with early conversion to psychosis in clinically high-risk individuals, and it is considered a risk factor of poor outcomes [4].

The idea that disturbances occurring early in brain development contribute to the pathogenesis of schizophrenia is often referred to as the neurodevelopmental hypothesis. International studies indicate that the prevalence of psychotic experiences in children is 7%. It has been suggested that environmental stress, socioeconomic status, maternal health conditions, and infections during pregnancy may affect the neurodevelopment of the fetus and lead to a vulnerability in the child to later stressors and psychopathology [39-41].

Protective factors against psychosis 

It has been speculated that both environmental and genetic factors have synergistic interaction in the initiation of psychosis. This complex interplay provides an insight into the role of a positive environment in protecting genetically susceptible individuals, particularly if these individuals are adolescents, as environmental factors are important at this stage [42-43]. Protective factors against psychosis can be categorized into three main groups: personal, family, and social.

In the study of psychosis, the role of the family environment, apart from the family history of psychosis, has received little attention. The impact of positive remarks and warmth from caregivers improved social functioning and symptoms in patients with psychotic prodrome [44]. Furthermore, there appears to be an adverse relationship between parental warmth and the contribution of genetic factors as the influence of genetic factors drop to almost zero in those that have received higher parental warmth [42].

Interestingly, family planning has also been found to modify an individual's psychiatric risk. A family of four children and less, with a gap of two years or more, has been found to have a protective effect. External familial support is of great value, and society plays a role. A good parenting system, clear boundaries at home, and a healthy relationship with siblings play a role in protecting against psychosis [45-46].

Attention has long been paid to exploring the role of personality in psychopathology. The five-factor model (FFM) or big five model consists of the following personality traits: neuroticism, extraversion, openness, agreeableness, and conscientiousness. Of all the personality traits, only neuroticism, which is characterized by vulnerability to emotional instability and self-consciousness, was positively associated with psychosis. Whereas the other four traits were considered negatively associated with psychosis and were, in fact, protective. Additionally, qualities like resilience are valuable for protecting against demanding environments [47-48].

A relatively higher intelligence quotient (IQ), higher neighborhood social cohesion, and positive home characteristics were found to be protective against psychosis, even among those exposed to multiple forms of victimization [49].

## Conclusions

Psychosis is a syndrome that is diagnosed by exclusion after secondary causes are ruled out. It is characterized by a disconnection from reality. Several genetic, environmental, and protective risk factors have been identified and require further study. The WHO considers psychotic disorders as a cause of disability. Psychotic disorders are a significant economic burden. Therefore, it is essential to try to detect the disease in its prodromal stage, where intervention may slow its progression and improve general wellbeing. Several tools have been identified to screen for prodrome, one of which is the PQ-B. This has been shown to be a promising tool that can be self-administered by the patient, in contrast to long interview-based tools, which are time-consuming and require a physician to perform. Despite the limited evidence, significant improvement has been found in the outcomes of patients with psychosis when they were treated in the prodromal period, especially with CBT. More trials should be undertaken to establish generalized guidelines for the treatment of psychosis in the prodromal stage.
